# Repeat ablation of atrial fibrillation using electrogram dispersion to identify additional areas of mechanistic significance

**DOI:** 10.1016/j.hroo.2024.07.007

**Published:** 2024-07-15

**Authors:** Junaid A.B. Zaman, Abdulhaseeb Khan, Jan Nielsen, Steen B. Kristiansen, Mads B. Kronborg, Christoffer T. Witt, Christian Gerdes, Jens Kristensen, Henrik K. Jensen, Peter Lukac, Sharad C. Agarwal

**Affiliations:** ∗ Keck School of Medicine, University of Southern California, Los Angeles, California; † Royal Papworth Hospital, Cambridge, United Kingdom; ‡ Department of Cardiology, Aarhus University Hospital, Aarhus, Denmark; § Department of Clinical Medicine, Aarhus University, Aarhus, Denmark

**Keywords:** Electrogram, Dispersion, Atrial fibrillation, Mapping, Termination, Repeat ablation, Biatrial

## Abstract

**Background:**

Electrogram dispersion identifies putative atrial fibrillation (AF) drivers in first time ablation procedures, with high acute termination rates and long-term outcomes akin to extensive ablation approaches. Its use in a population that had undergone repeat ablation is unknown, particularly where the pulmonary veins are already isolated.

**Objective:**

This purpose of this study was to assess electrogram dispersion mapping during repeat ablation procedures for persistent AF.

**Methods:**

One hundred sixty-seven patients from the United Kingdom and Denmark, all with persistent AF recurrence after prior ablation procedure(s), were mapped using a five splined catheter for electrogram dispersion before ablation. Areas were manually tagged on biatrial electroanatomic maps and ablated once pulmonary vein isolation was confirmed or reisolated if required. All patients had 12-month continuous monitoring, with most of the cohort having follow-up beyond 24 months.

**Results:**

Of the 167 patients [53 (32%) female; mean age 66 ± 8 years; mean left atrial (LA) diameter 4.8 cm; mean ejection fraction 53%], 108 had pulmonary veins already isolated. Dispersion sites occurred in both atria (3.2 LA, 1.4 right atrium). Acute termination to sinus rhythm occurred in 71 (42%) of the cohort patients, with a further 73 (44%) terminating to atrial tachycardia/flutter. At 12-month follow-up, 95% of patients were free of AF, with 74% overall freedom from all atrial arrhythmias. Heart failure and severely enlarged LA predicted recurrence, and termination to sinus improved freedom from all atrial arrhythmias.

**Conclusion:**

Dispersion mapping is a promising approach at repeat ablation procedures for persistent AF, with high acute termination rates and good clinical outcomes. Further prospective randomized trials are needed to evaluate this approach in a population that had undergone repeat ablation.

**Key Findings**

▪ Ablating areas of electrogram dispersion at repeat ablation led to 95% freedom from atrial fibrillation (AF) at 12 months in 2 international academic centers.
▪ The presence of preexisting pulmonary vein (PV) isolation did not improve outcomes compared to those needing PV reisolation.
▪ The absence of AF termination or organization predicted clinical failures, with those terminating to sinus rhythm having the best outcomes.
▪ Dispersion areas lay in both right and left atria.

## Introduction

The improvement in outcomes from atrial fibrillation (AF) ablation over the last few decades can be attributed to better technologies for ablation energy delivery, lesion titration, and more objective verification of procedural end points. This is evident in the increase in pulmonary vein isolation (PVI) success rates in randomized controlled trials,^1^ leading to an increased level of recommendation in recent guidelines.^2^

However, 1 potential ceiling to this improvement is the mechanistic understanding of persistent AF. This has focused on pulmonary vein (PV) antra for first time ablation, with additional ablation strategies at first ablation having no benefit in data in initial randomized controlled trials.^3^, ^4^, ^5^ Recent addition of low voltage,^6^ linear ablation,^7^ and vein of Marshall^8^ has shown improvement over PVI-only results, stimulating discussion and further trials in the “PVI +” approach at first ablation.

The focus on initial single-procedure approaches does not address the fact that many patients need repeat ablation of persistent AF. Studies in this population show worse outcomes, often related to low-voltage areas,^9^ with the role of reconnected veins in recurrence mechanisms debated.^10^ The systematic study of mechanisms of recurrence is important in knowing how to address substrates both at the PV antra and beyond, which may improve success from repeat ablation procedures.^11^

One such approach is mapping AF regions of interest for dynamic substrates, rather than empirical fixed lesion sets. An increasingly adopted technique is electrogram dispersion,^12^^,^^13^ with recent data on persistent and long-standing persistent first time ablation.^14^, ^15^, ^16^ However, this approach has not been studied in populations that had undergone repeat ablation, which motivated our study.

We set out to study outcomes from repeat persistent AF ablation procedures using electrogram dispersion in 2 international centers using this approach—Denmark and the United Kingdom. This was a single-arm, nonrandomized, retrospective cohort study. Institutional review board approval was obtained for both sites and data analysis at the third site—the United States.

## Methods

**Figure 1:**
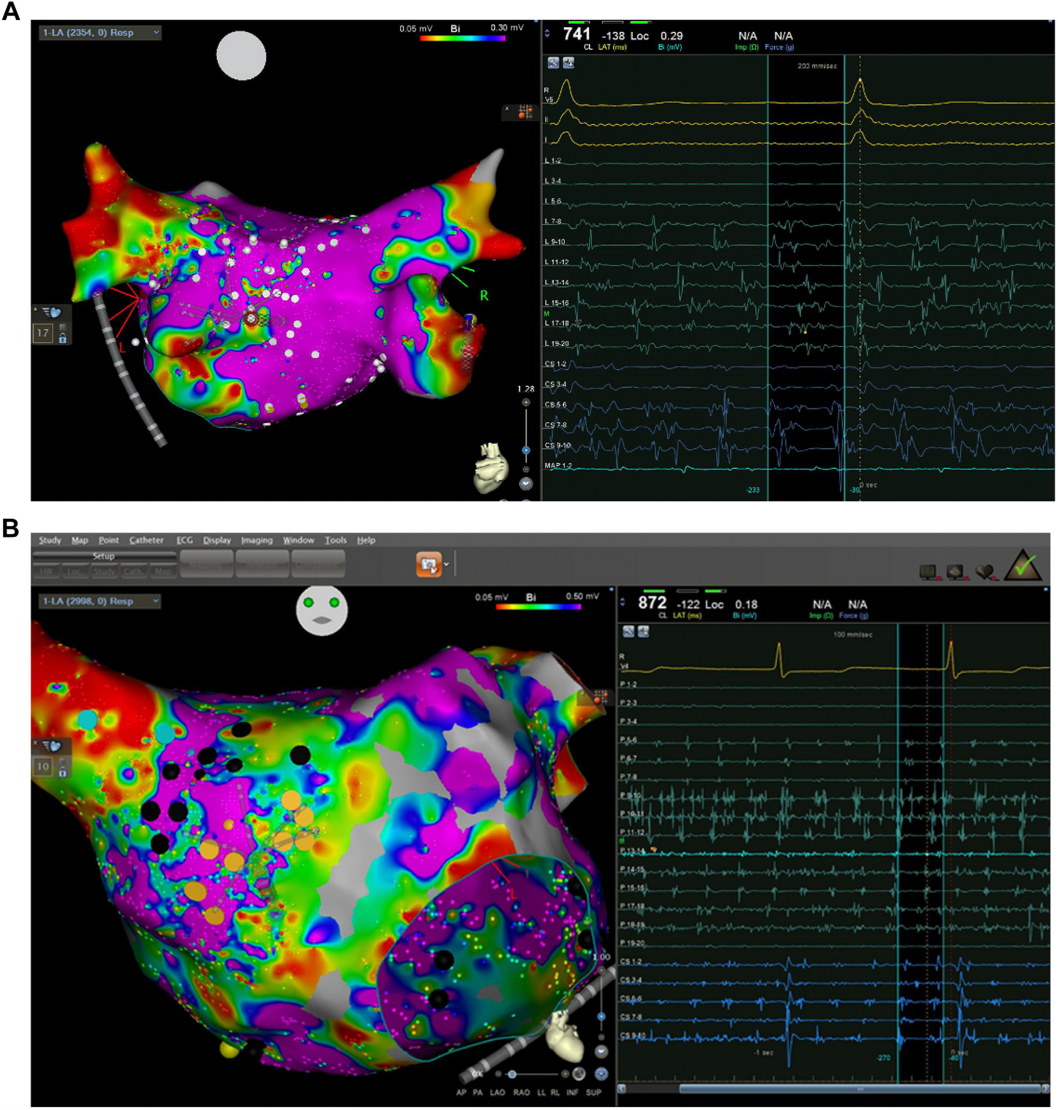
Examples of electrogram dispersion annotation from this study. **A:** Posterior wall left atrial (LA) dispersion sites in *white* in a patient with reconnection of the right pulmonary veins. **B:***Black tags* anterior to the right superior pulmonary vein in a patient reflect numerous cycles of spatiotemporal dispersion on the PentaRay catheter in a patient with pulmonary veins isolated.

Between 2018 and 2022, 167 consecutive patients underwent repeat ablation for persistent AF. Antiarrhythmics were held for 5 half-lives, or a week if on amiodarone. After exclusion of clot as per local practice guidelines, transseptal puncture was performed. A PentaRay (Biosense Webster, Irvine, CA) catheter was used to assess PVs, baseline voltage including areas of dispersion (Figure 1). All research reported has adhered to relevant ethical guidelines and is in compliance with local institutional review boards on human studies, including obtaining patient consent.

Electrogram dispersion was annotated using same criteria as the original paper,^12^ without automated software by the primary operator (Figure 1). If dispersion sites lay close to PVI lesion sets, extension of those lesion sets to incorporate was performed as part of any repeat PVI. Once PVI was confirmed, all areas of dispersion identified on the initial map were ablated using clustering of lesions or connecting to the adjacent nonconducting boundary.

If the rhythm converted to atrial tachycardia (AT)/atrial flutter (AFL), this was mapped and ablated using standard techniques in CARTO (Biosense Webster, Irvine, CA). If converted to sinus, a reinduction protocol was performed to induce any further AF or AT, which was then mapped using the above methods.

This process was repeated in the right atrium (RA) if there was no acute rhythm impact from left atrial (LA) dispersion ablation. If patient remained in AF at the end of the procedure, they were cardioverted.

Clinical follow-up was as per local institutional policy, with a minimum of 48-hour Holter monitoring and most often 5-day Holter monitoring at 12 months in all patients. Electrocardiography was done at 6 and 12 months. If patients had symptoms in the interim period or beyond 12 months, further Holter monitoring was done, which was based on clinical symptoms. Because of the primary care national databases in Denmark and the National Health Service General Practioner network in the United Kingdom, all patients had clinical assessment during beyond 12-month follow-up.

### Statistical methods

Categorical variables are expressed as count (percentage) and numerical variables as mean ± SD. Categorical variables were compared using the χ^2^ test or the Fisher exact test, as appropriate. Nominal variables were compared using the Student *t* test, as appropriate. Kaplan-Meier survival curves were assessed and compared using the log-rank test. The outcome is unknown in patients lost to follow-up and in patients who died during follow-up. In all such cases, the time of follow-up was recorded and interpreted as censored data. A 2-sided *P* value of <.05 was used to indicate statistical significance. Multivariate Cox regression analysis was used to analyze effects between significant variables. All statistical analyses were performed using R version 43.2 software.

## Results

From 2018 to 2022, 167 patients were enrolled and underwent repeat ablation with electrogram mapping; 53 (32%) were female, with a mean age of 66 ± 8 years. Forty-two patients had undergone >1 ablation procedure previously. Patients had a mean LA diameter of 4.8 cm, with 19 (11%) having a highly enlarged LA (>72 mL and >52 mm), 88 (53%) having a moderately enlarged LA (52–72 mL and 41–52 mm), and 60 (36%) had a nonenlarged LA (<52 mL and 40 mm). The mean ejection fraction was 53% ± 10%. Eighty-two (49%) had a history of hypertension and 19 (11%) had a history of diabetes. Twenty-eight patients (17%) were taking amiodarone at the time of the procedure. The baseline characteristics of all enrolled patients, including medication before enrollment, are presented in Table 1.

**Table 1:** Baseline characteristics of the study population (N =167)

Characteristic	Value
Age (y)	66 ± 8
Female	53 (32)
HTN	82 (49)
DM	19 (11)
Medications	
β-Blocker	120 (72)
Amiodarone	28 (17)
Class 1c	8 (5)
Ejection fraction (%)	53 ± 10
LA size	
Normal (<52 mL and <40 mm)	60 (36)
Moderately enlarged (52–72 mL and 41–52 mm)	88 (53)
Highly enlarged (>72 mL and >52 mm)	19 (11)
AF subtype	
Persistent	161 (96)
Long-standing persistent	6 (4)

Values are presented as mean ± SD or n (%).

AF = atrial fibrillation; DM = diabetes mellitus; HTN = hypertension; LA = left atrium.

### Ablation procedure

**Figure 2:**
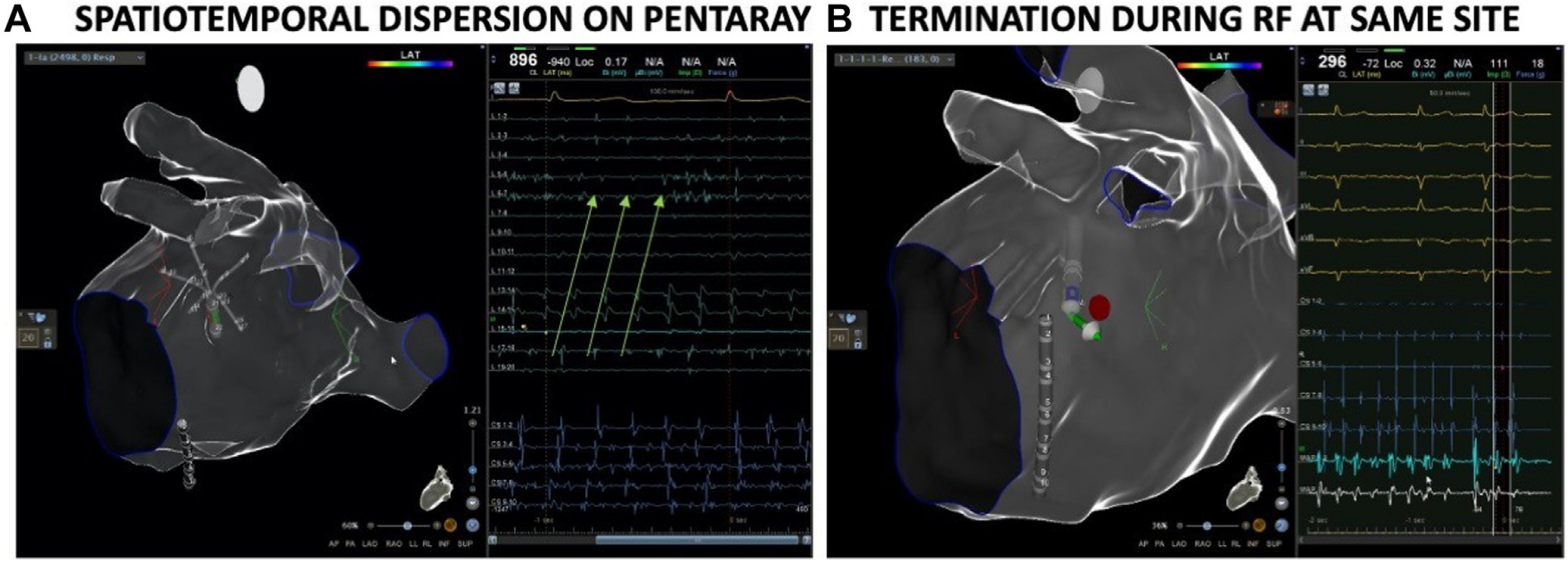
Acute impact of dispersion area ablation. (**A**) Example of electrogram dispersion inferior to left inferior pulmonary vein (3 cycles annotated with *arrows*) where (**B**) atrial fibrillation terminated to sinus during ablation at this area.

The mean total procedure time, including anesthesia and transesophageal echocardiogram, was 266 ± 80 minutes, with a mean ablation time of 29 ± 20 minutes. PVI was already present in 108 patients (64%). All patients underwent LA mapping, and 75 (45%) underwent additional RA mapping. Patients had a mean of 3.2 LA and 1.4 RA dispersion sites. The total number of ablation sites in patients with and without preexisting PVI did not have a statistically significant difference, with a mean of 4.1 ± 2 and 3.6 ± 1, respectively. In addition, the mean number of LA and RA ablation sites did not differ significantly between patients with and without PVI. AF terminated in 144 patients (86%), with direct conversion to sinus rhythm obtained by ablation in 42% of the patients (Figure 2). In patients where conversion to sinus rhythm was not achieved, 23 (14%) had no acute effect, 25 (15%) converted to AT, and 43 (26%) converted to AFL. Patients who converted to sinus rhythm had a mean procedure time of 243 ± 70 minutes, while those who did not had a mean procedure time of 282 ± 83 minutes (Table 2).

**Table 2:** Procedure details

Characteristic	Value
Total procedure time (min)	266 ± 80
Ablation time (min)	29 ± 20
PVI	108 (64)
LA mapped	168 (100)
RA mapped	75 (45)
LA ablation sites	3 ± 1
RA ablation sites	1 ± 1
Termination rhythm	
Sinus	70 (42)
AT	25 (15)
AFL	43 (26)
Other	6 (4)
No effect (AF)	23 (14)

Values are presented as mean ± SD or n (%).

AF = atrial fibrillation; AFL = atrial flutter; AT = atrial tachycardia; LA = left atrium; PVI = pulmonary vein isolation; RA = right atrium.

### Clinical outcomes

**Figure 3:**
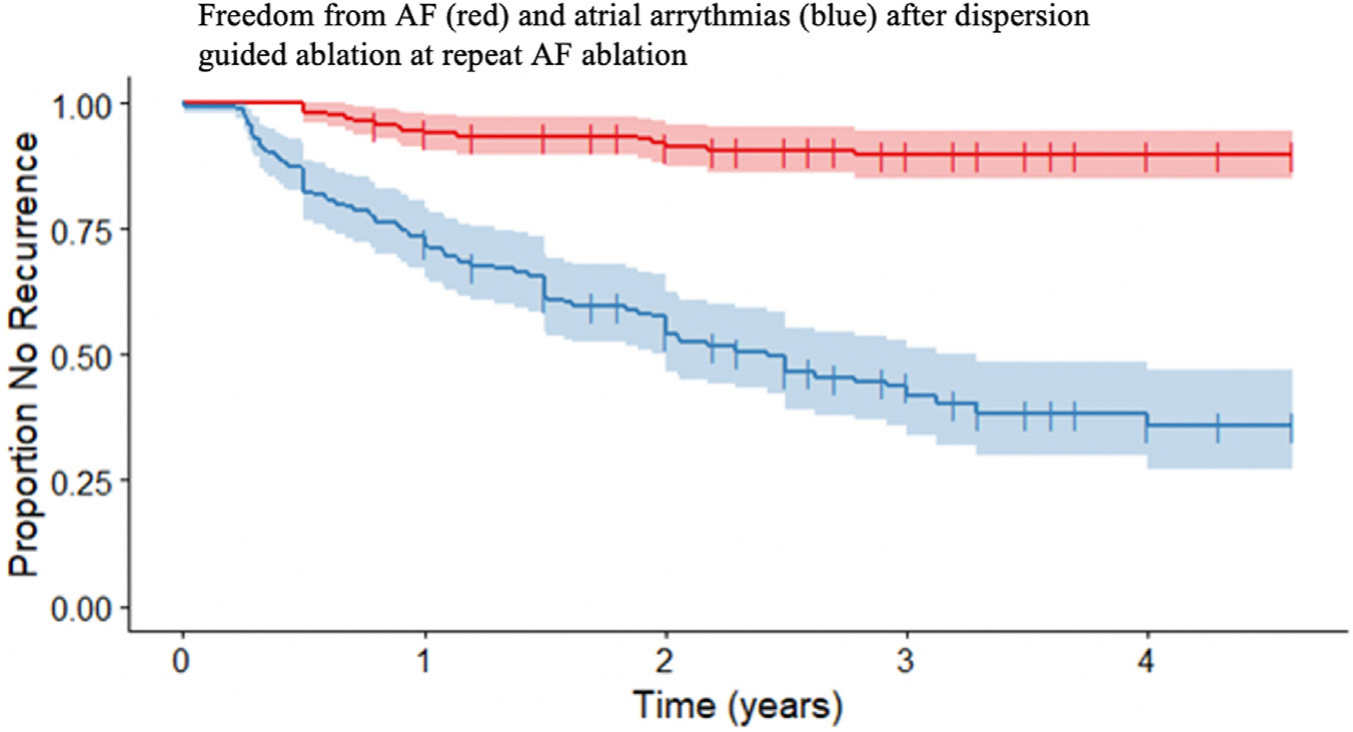
Overall atrial fibrillation (AF)– and arrhythmia-free survival after 1 dispersion-guided repeat ablation procedure. Kaplan-Meier curves showing the proportion of the study population with a recurrence of AF (*red*) and all atrial arrhythmias (*blue*) during the total follow-up period. Censored values (+) indicate the last known follow-up time for those patients who had no recurrence.

One hundred sixty-six (99.4%) in the study population completed 12-month follow-up. One patient died 3 months before the end of the first year of follow-up (heart failure exacerbation). Patients were followed for a mean of 2.92 ± 0.9 years, with 159 patients having follow-up beyond a year and 116 patients beyond 2 years. At 12 months, after 1 dispersion guided repeat ablation procedure, 159 patients (95%) were AF free, and 124 (74%) remained free of atrial arrhythmia (Figure 3). Of the patients with recurrences, 8 (5%) had AF, with 35 (21%) with a recurrence of AT or AFL. Beyond 12 months, a total of 10 patients (6%) had a recurrence of AF, 45 recurred with AFL (27%), and 3 (2%) recurred with AT (Table 3). One hundred forty-seven patients (88%) were found to be in sinus rhythm at their last documented follow-up visit. In total, 23 patients (14%) had a further ablation procedure, 21 for AFL and 2 for AF (of which 1 chose an ablate and pace strategy). A further 14 (8%) underwent cardioversion during follow-up.

**Table 3:** Outcomes of repeat ablation at 12 mo

Characteristic	Value
AF	8 (5)
AT	2 (1)
AFL	33 (20)
No recurrence	124 (74)

Values are presented as n (%).

AF = atrial fibrillation; AFL = atrial flutter; AT = atrial tachycardia.

### Predictors of recurrence

**Figure 4:**
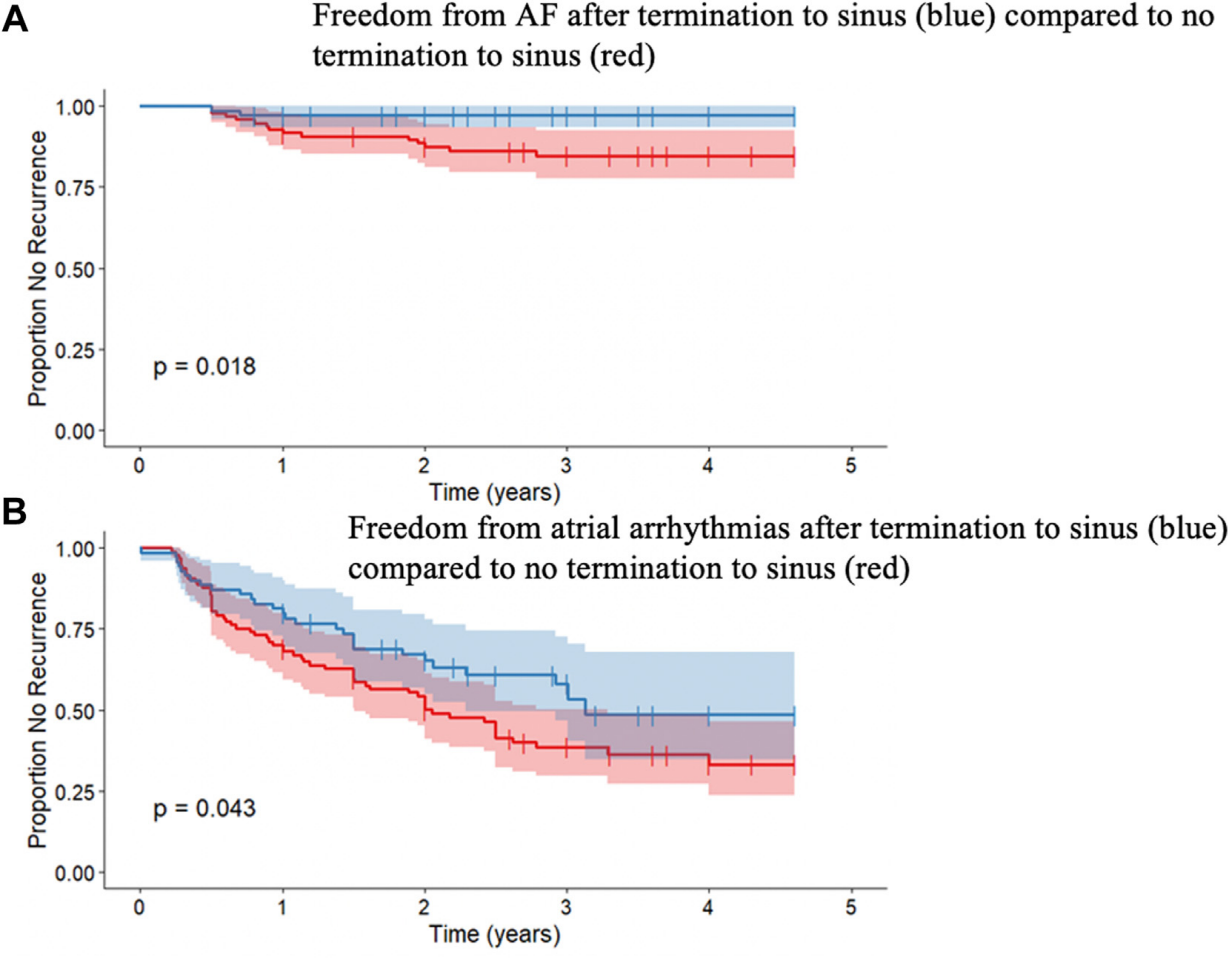
Freedom from atrial arrhythmias in the sinus termination subgroup is improved. **A:** Kaplan-Meier curves showing the proportion of the study population with a recurrence of atrial fibrillation without termination to sinus (*red*) and with termination to sinus (*blue*) during the total follow-up period. **B:** Kaplan-Meier curves showing the proportion of the study population with a recurrence of atrial arrhythmia without termination to sinus (*red*) and with termination to sinus (*blue*) during the total follow-up period. Censored values (+) indicate the last known follow-up time for those patients who had no recurrence. *P* value is calculated using the log-rank test.

**Figure 5:**
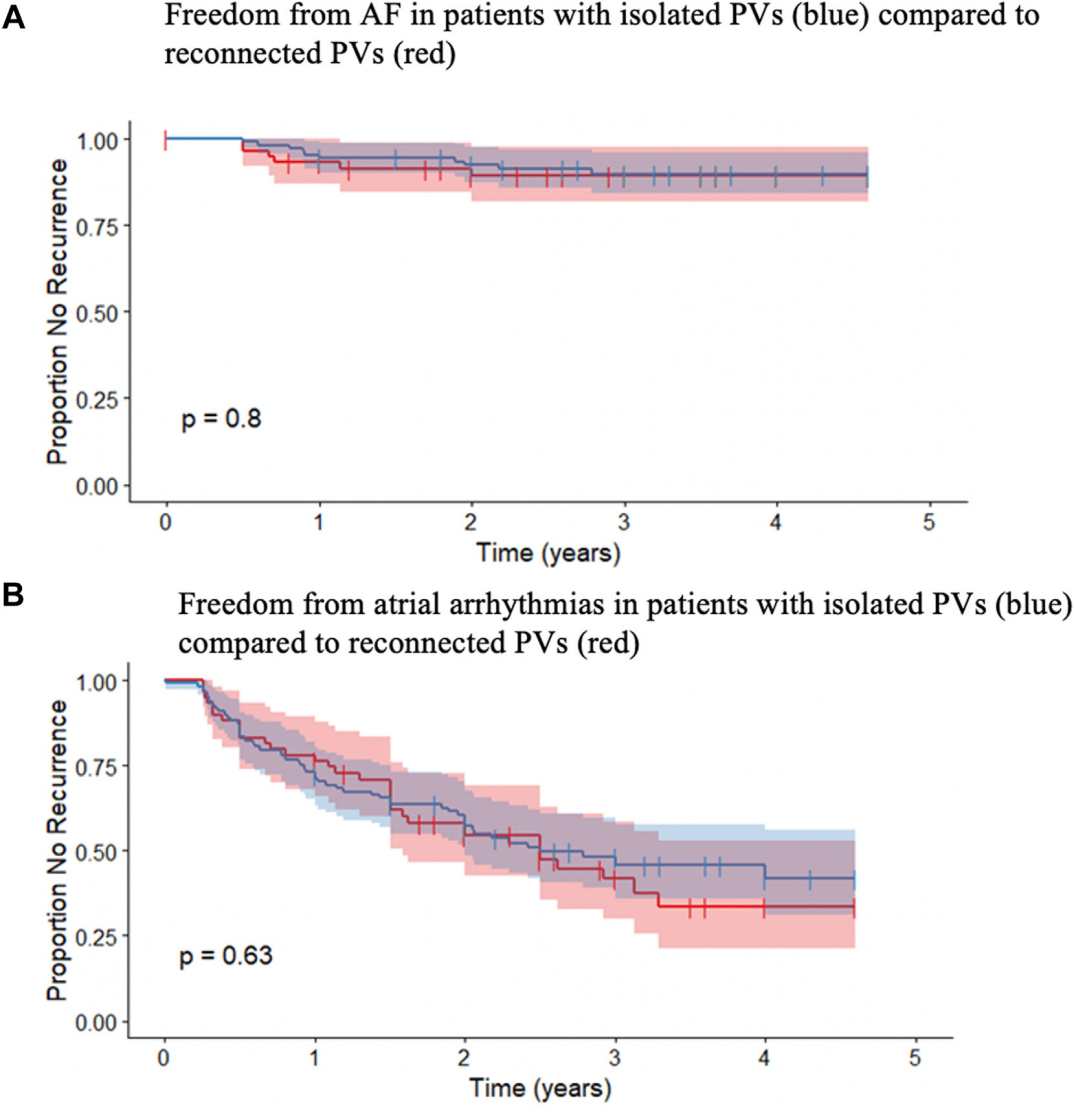
Freedom from atrial arrhythmias in patients with and without pulmonary veins already isolated. **A:** Kaplan-Meier curves showing the proportion of the study population with a recurrence of atrial fibrillation without pulmonary vein isolation (PVI) (*red*) and with PVI (*blue*) during the total follow-up period. **B:** Kaplan-Meier curves showing the proportion of the study population with a recurrence of atrial arrhythmia without PVI (*red*) and with PVI (*blue*) during the total follow-up period. Censored values (+) indicate the last known follow-up time for those patients who had no recurrence. *P* value is calculated using the log-rank test.

Patients who had a recurrence of AF had a mean age of 62 ± 10 years and did not differ significantly from those who did not recur (66 ± 8; *P* = .25). The percentage of male and female patients who had recurrence of AF did not differ significantly at 1.9% and 6.5%, respectively (*P* = .40). Patients with a highly enlarged LA (>72 mL and >52 mm) were shown to have a significant association with recurrence of AF (*P* = .04871) (Online Supplemental S1). Conversion to sinus rhythm by ablation was shown to have a statistically significant association with decreased recurrence of AF (Figure 4A) and atrial arrhythmias (Figure 4B) when compared to those converted to nonsinus rhythm (AT, AFL) or in whom ablation had no effect (*P* = .0479). In addition, patients with a known history of heart failure had a statistically significant association with recurrence of atrial arrhythmia (*P* = .04288). A history of diabetes and hypertension did not demonstrate an association with recurrence. The total number of sites ablated and RA ablation did not have a statistically significant association with recurrence of AF or atrial arrhythmias. The procedure time did not differ significantly in patients who had recurrences of AF and those who did not, with means of 260 ± 106 and 266 ± 80, respectively. In addition, PVI from the previous procedure did not have a statistically significant association with recurrence of AF (*P* = .45) or atrial arrhythmias (*P* = .14) (Figures 5A and 5B). In multivariate analysis, the only requirement for cardioversion at the end of the procedure was associated with increased risk of recurrence of AF (*P* = .0003) and all atrial arrhythmias (*P* = .022).

## Discussion

The main findings of this study are follows: (1) a 95% of freedom from AF using a dispersion approach at 12 months; (2) these results are maintained in the large subgroup of patients with PVs already isolated; (3) the absence of AF termination predicted clinical recurrence; (4) patients with termination to sinus rhythm had better outcomes at 12 months; and (5) recurrent arrhythmias were mainly organized rhythms such as atrial flutter.

### PVI at repeat ablation procedures

The “PVs already isolated, yet recurrent AF” patient is increasingly common and arguably will be more so with the advent of newer technologies and improved first-pass PVI rates. Our data are in keeping with prior studies looking at reconnection,^17^ in that up to half of patients will have some reconnection at repeat ablation. In these patients, the clinical judgment of whether the recurrent AF can all be attributed to the often small reconnection leads to operators searching for additional substrate. Our data shed light on this important patient group, by demonstrating that mapping and ablation of electrogram dispersion can improve outcomes in a challenging subgroup even in latest studies.^9^^,^^18^, ^19^, ^20^, ^21^, ^22^

Importantly, many of the dispersion areas lay remote to the reconnection sites, confirming an independent role in the mechanisms of AF recurrence, and further evidenced by termination occurring at the dispersion site, after ablation had no impact at the PV reconnection site.

### Dispersion areas and relation to AF mechanisms

Areas displaying dispersion were present in both atria, with an ∼3:1 left to right ratio. This is similar to other AF mapping studies that have studied both atria.^23^ They also lay outside any reproducible anatomic locations, suggestive of a dynamic substrate and reducing the chances of incidental ablation with empirical lines. Mechanistically, dispersion of electrograms in AF may be akin to spanning a whole tachycardia cycle length for a localized reentry atrial tachycardia (AT), and previous work has suggested colocalization of AT mechanisms at repeat ablation with previous AF driver sites.^9^^,^^24^ This suggests that the presence of dispersion itself may be a marker of a more organized form of AF, comprising localized reentry regions which owing to competing and interacting regions result in a fibrillatory conduction pattern and with ablation can organize into coherent rhythms such as AT and atrial flutter.^25^ In the original study by Seitz et al,^12^ dispersion was related to spiral waves in numerical studies, and this is a source of potential underlying agreement with other mapping systems, but also one of semantic disagreement as the required definitions of spiral wave activity (or “rotors”) cannot be proven with clinical recording systems. One possibility, supported by this study is that dispersion is a useful surrogate of a dynamic localized organized conduction pattern.

### Mode of termination and relation to outcomes

The above mechanistic model of competing areas of local AF organization would be expected to lead to progressive organization of AF as the sites are treated, and the eventual conversion to a coherent rhythm such as AT or flutter. This paradigm does not fit with sinus rhythm termination, but previous studies have shown the mechanisms by which a spiral wave or focal source may abruptly terminate to sinus rhythm on the basis of local conduction properties.^26^ Also, the mechanisms by which spiral waves can abruptly disintegrate and hence allow return of sinus rhythm (given adequate sinus rates) have been shown to be stochastic in nature, rather than truly a linear progression.^27^ Whether this is in part responsible for the much higher sinus rhythm termination rates with dispersion mapping is beyond the scope of these data to address, but the increased freedom from AF in the sinus rhythm termination group suggests that research focus should be on these sites in particular, as they contain hierarchically important mechanisms, and not just bystander locally organized sites. Sinus rhythm termination has also been shown to confer improved freedom in a meta-analysis of all AF driver ablation studies^28^ and in previous comparison studies.^29^ This is also shown by the abrupt extinguishing of wavelets with cardioversion not adequately treating underlying mechanisms with the increased recurrence rates in this subgroup of this study.

### Incorporating dispersion mapping into the clinical workflow

There are challenges incorporating any new mapping technique into routine clinical practice, and the mapping time preablation is longer when electrograms are studied in detail for multiple cycles. We have found that organizing the review screen with radial PentaRay electrodes adjacent (A3,4 next to B3,4 rather than A1,2) can help accelerate the detection of these patterns visually. This is also a reason why initial cases with the Octaray were of no additional benefit in visually detecting these areas, as there were too many electrograms to reorder and an overall direction of propagation was less easy to visualize. One additional factor is that the best dispersion maps are obtained with all splines of the catheter equally spaced, which is more difficult to achieve with an Octaray, especially on the anterior wall. If a patient has documented previous PVI and confirmation of posterior wall and anterior line isolation, it may also be beneficial to perform RA mapping first before transseptal puncture, which can plan the workload of the case better than leaving it until the end of LA ablation to discover numerous targets in the RA. From comparison with other AF mapping methods in the literature, dispersion mapping does not add significant time, as areas are acquired as part of initial electroanatomic mapping and are shorter to analyze than the 30 seconds required by CARTOFINDER. The number of cycles of remapping and the choice of the end point, especially in patients who do not have termination of AF, are a subject of ongoing research.

### Limitations

The first major limitation of all repeat ablation studies is that there is no comparison performed to another repeat ablation strategy. Similarities between PV isolated and PV reconnected subgroups and the lack of a “gold standard” approach for repeat ablation formed the rationale behind this, and a “PVI touch-up approach only” is not a suitable comparator, as this is not the main strategy in either center. This is due to the mechanistic disparity between a persistent AF substrate at redo ablation and a single PV reconnection, often in a vein that has little activity. A PVI vs PVI + dispersion randomized controlled trial for first time ablation is already enrolling, with substudy results published.^15^ Second, there was no automated approach to dispersion mapping available at the time this was conducted, which is now available (VX1, Volta Medical, Marseille, France).^14^^,^^15^ This added procedure time and interoperator variability to the areas annotated, but during retrospective case review, similar electrogram features were highlighted by each site’s main operator (S.C.A., P.L.). A third limitation is the end point of dispersion ablation if the rhythm did not terminate or organize in the LA. All such patients underwent RA mapping, but the number of cycles of remapping and ablation of dispersion sites was left to operator discretion. Finally, these studies were performed using PentaRay, not Octaray. However, all patients were studied using the same catheter and this was the same as the original description in 2017. Of note, the automated software can be used with any high-density catheter.

## Conclusion

In this study of repeat ablation procedures for persistent AF, the use of electrogram dispersion mapping and patient-specific ablation strategies led to high freedom from AF during long-term follow-up. This study shows the potential for mapping AF real time with a widely used commercial catheter and also showed that a biatrial approach was needed in many patients to fully assess the mechanistic sites of importance. We conclude that this is a promising approach, with clinical utility and minimal impact on the workflow in an often challenging patient population.
