# Supplementary material for: Repeat ablation of atrial fibrillation using electrogram dispersion to identify additional areas of mechanistic significance
Source: Heart Rhythm O2. 2024 Jul 15;5(8):543–50. doi: 10.1016/j.hroo.2024.07.007 (PMC11385402; doi:10.1016/j.hroo.2024.07.007)
Supplement: Table S1 [file mmc1.docx]

**Table S1: Association of LA Size and Recurrence of AF**

| **LA Size** | **Recurrence of AF** | **No Recurrence of AF** |
| --- | --- | --- |
| Highly Enlarged (>72ml & >52mm) | 3 | 16 |
| Normal (<52 ml & <40mm) or Moderately Enlarged (52-72 ml & 41-52mm) | 5 | 143 |

Values are n

AF = Atrial Fibrillation
